# Supplementary material for: Anti-α-Glucosidase and Antiglycation Activities of α-Mangostin and New Xanthenone Derivatives: Enzymatic Kinetics and Mechanistic Insights through In Vitro Studies
Source: Molecules. 2022 Jan 15;27(2):547. doi: 10.3390/molecules27020547 (PMC8777799; doi:10.3390/molecules27020547)
Supplement: Supplementary file 1 [file molecules-27-00547-s001.zip › molecules-1544743-supplementary.pdf]

Anti-α-Glucosidase and Antiglycation Activities of α-Mangostin and New Xanthenone Derivatives: Enzymatic Kinetics and Mechanistic Insights Through In Vitro Studies

Francine Medjiofack Djeujo [1], Valeria Francesconi [2], Maddalena Gonella [1], Eugenio Ragazzi [1], Michele Tonelli [2,*], and Guglielmina Froldi [1,*]

[1] Department of Pharmaceutical and Pharmacological Sciences, University of Padova, 35131 Padova, Italy; francine.medjiofackdjeujo@phd.unipd.it (F.M.D.), maddalenagonella96@gmail.com (M.G.), eugenio.ragazzi@unipd.it (E.R.)

[2] Department of Pharmacy, University of Genova, 16132 Genova, Italy; francesconi.phd@difar.unige.it

* Correspondence: g.froldi@unipd.it, Tel.: +39-049-827-5092; Fax: +39-049-827-5093 (G.F.); tonelli@difar.unige.it (M.T.)

Supplementary materials

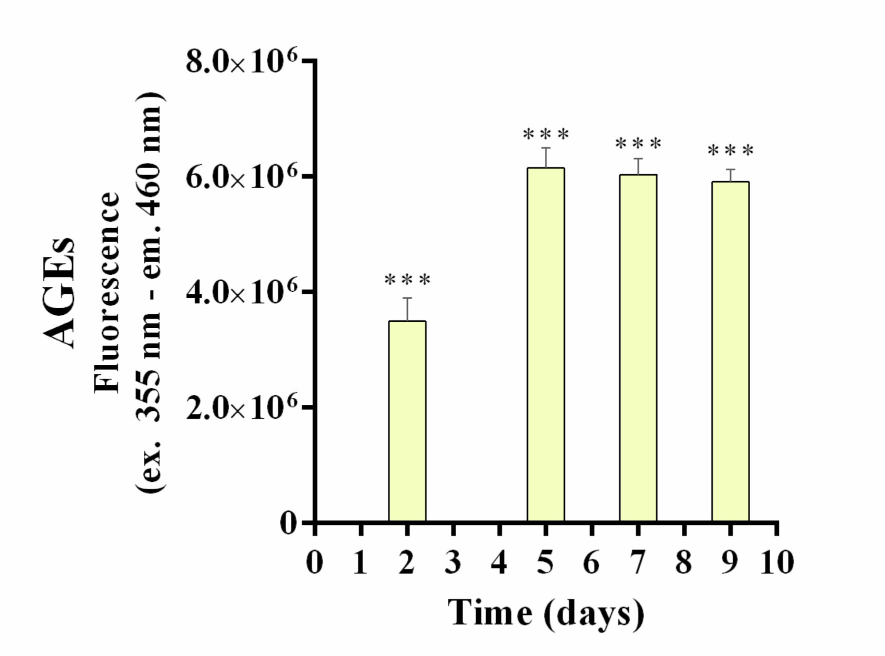

Figure S1 AGE formation after 2, 5, 7 and 9 days of incubation of 50 mg/mL BSA with 0.1 M ribose. *** p<0.001 *vs* AGEs formation without ribose (controls).

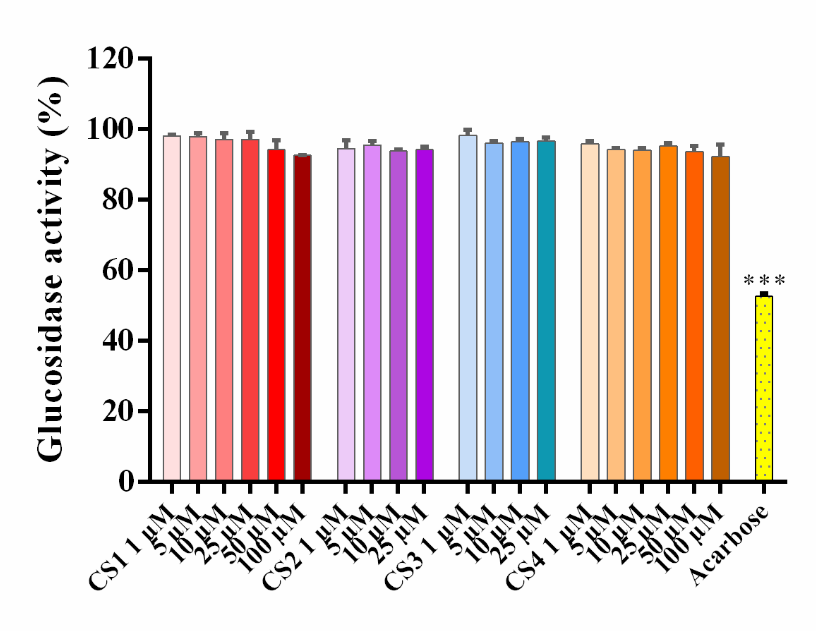

Figure S2 Effects of synthetic compounds CS1-CS4 on 0.04 µM α-glucosidase activity. Acarbose concentration: 1.25 mM. *** p<0.001 vs α-glucosidase activity without inhibitor (controls).
